# Abundance of *Yersinia pestis* Nucleic Acids in Soil from *Rattus tanezumi* Plague Foci in Yunnan Using ddPCR

**DOI:** 10.3390/pathogens15060616

**Published:** 2026-06-09

**Authors:** Yongmei La, Fan Li, Cunjuan Duan, Jinjiao Kong, Haipeng Zhang, Hongli Tan, Baoxiang Li, Youhong Zhong, Shilong Yang, Peng Wang, Liyuan Shi

**Affiliations:** 1 School of Public Health, Dali University, Dali 671000, China; 15699397099@163.com (Y.L.);; 2 Yunnan Provincial Key Laboratory of Zoonosis Control and Prevention, Yunnan Institute of Endemic Diseases Control and Prevention, Kunming 650500, China; m18856164010@163.com (F.L.); 15393954571@163.com (C.D.);; 3 School of Public Health, Kunming Medical University, Kunming 650500, China; 4 Longchuan County Centre of Diseases Control and Prevention, Dehong Dai and Jingpo Autonomous Prefecture 678700, China

**Keywords:** *Yersinia pestis*, soil, droplet digital PCR (ddPCR)

## Abstract

To determine whether *Yersinia pestis* (*Y. pestis*) signals were present in the soil, 78 soil samples were collected from 10 counties identified as *Rattus tanezumi* plague foci and another six counties in non-foci areas in Yunnan, China, from August 2024 to May 2025. Nucleic acids were extracted using the DNeasy PowerSoil Pro Kit (Qiagen) and detected with a Bio-Rad QX200 droplet digital PCR (ddPCR) system targeting two *Y. pestis* genes (*caf1* and *ypo0392*). Our sampling design also considered two additional dimensions: nine soil types and three habitat types. The raw data obtained from ddPCR were copy numbers. A gene was considered positive when its copy number exceeded the limit of detection (LoD). A sample was considered positive if the copy number of *ypo0392* exceeded its LoD (regardless of *caf1*), or if both genes exceeded their respective LoDs. No correlation was detected between the positivity rate of *Y. pestis* and the division of sampling sites into plague focus and non-focus regions (*p* = 0.758). Similarly, the Mann–Whitney U test revealed non-significant differences in pathogen copy number across the two site categories, with *p* = 0.603 for the *caf1* gene and *p* = 0.372 for the *ypo0392* gene. No statistical difference in positivity rate was found for either soil types or habitat types. However, for both the grouping variables, a statistical difference in copy number was observed. Our results indicated that quantifying nucleic acid abundance by copy number provided richer information than a simple positive/negative determination. The detection of *Y. pestis* signals was associated with soil type, rather than with whether an area was classified as a focus or non-focus region. Accordingly, future research on the preservation mechanism of *Y. pestis* should not be restricted to the concept of natural foci but should adopt a broader perspective.

## 1. Introduction

Yunnan, China, is considered the place of origin for the Third Plague Pandemic [1]. Strains isolated from the natural plague foci of *Rattus tanezumi* (commonly known as the domestic rat plague focus) in residential areas in mountainous western Yunnan and coastal Fujian and Guangdong predominantly belong to the 1.ORI2 genotype [2]. These strains are the descendants of the bacteria that colonized Yunnan after being introduced during the Third Plague Pandemic [3].

Plague in the *Rattus tanezumi* plague focus in Yunnan exhibits a pattern of alternating epidemics and quiescence. Since the 1950s, two major epidemic periods have occurred: the first from 1950 to 1956, followed by 26 years of quiescence, and the second from 1982 to 2007. Since 2007, the focus has remained largely quiescent, with only sporadic cases reported in 2016 [4,5] and 2020 [6]. However, the mechanisms underlying this epidemic–quiescence cycle remain unclear. For a focally transmitted natural disease, such as plague, determining where the plague pathogen persists during quiescent periods and understanding its state of persistence and distribution in the environment are crucial scientific questions for elucidating the maintenance mechanisms of the focus.

The domestic rat plague foci in Yunnan are primarily distributed across the Hengduan Mountains in western Yunnan, the southern border area, and the central valley regions, covering 46 counties (districts) within 11 prefectures (cities) at elevations ranging from 400 to 2100 m [7]. Since 1950, the epidemic has shown a wavelike spread from border areas to the interior and from mountainous regions to towns. This spatial transmission dynamic is closely related to the region’s vertically zoned ecological characteristics. It has been reported that soils from three plague foci in Yunnan, China were acidic, slightly saline, and enriched in several metals, with properties varying significantly by region [8], suggesting that soil may play an important role in the natural plague niche. Although surveillance of host and vector populations in foci has been conducted long-term, research on the focal habitat, particularly concerning environmental soil, remains relatively scarce.

Through experiments, it was found that *Y. pestis* biotype Orientalis could remain viable and fully virulent after 40 weeks in soil [9], and studies have reported the isolation of *Y. pestis*-specific phages from soil samples [10,11]. However, due to the extremely low concentration of *Y. pestis* in the environment, both conventional bacterial culture and nucleic acid amplification via PCR face significant technical challenges. The natural presence of PCR inhibitors in soil, combined with the typically low concentration of target substances in environmental samples, significantly reduces the detection rate of target nucleic acids [12]. In recent years, the emergence of digital PCR (ddPCR) has provided a powerful tool for the highly sensitive detection and absolute quantification of pathogen nucleic acids in environmental samples. This technology enables the detection of nucleic acids at the single-copy level, thereby providing a technical possibility for identifying trace amounts of *Yersinia pestis* nucleic acids in environmental samples [13,14].

In this study, soil nucleic acid (DNA) from Yunnan Province was selected as the research object. The sampling design took into account both domestic rat plague foci and non-foci, as well as different soil types and habitat types. A duplex droplet digital PCR (ddPCR) assay targeting two genes (*caf1* and *ypo0392*) was employed in this study to detect *Yersinia pestis* nucleic acids in soil samples, aiming to address the following scientific questions: (1) Whether there is a correlation between the distribution of *Yersinia pestis* nucleic acids in the current environment and historical plague prevalence at sampling locations; and (2) whether different soil types and habitat types affect the distribution characteristics of *Yersinia pestis* nucleic acids in the environment. This study will provide insights into the maintenance mechanism of the endemicity of plague foci and precise environmental monitoring strategies.

## 2. Materials and Methods

### 2.1. Sample Sources and Variable Definitions

From August 2024 to May 2025, soil samples were collected from 10 counties identified as *Rattus tanezumi* plague foci, and from six counties identified as non-foci areas in Yunnan Province. For each sampling area, 1–10 sampling sites were set up (with an attempt to cover various habitat types). At each sampling site, three parallel soil samples were collected at intervals of 5 m, with a sampling depth of 30 cm. The collected samples were placed in 50 mL centrifuge tubes and properly preserved in a portable refrigerator at −20 °C. After being transported back to the laboratory, the three parallel samples from each sampling site were pooled into one final sample and thoroughly homogenized, which was then used for subsequent nucleic acid extraction.

This study took soil from plague foci in Yunnan Province as the main research object, and used soil from non-plague foci as a control. Therefore, sampling covers both plague foci and non-plague foci. We refer to this as the epidemiological type variable.

According to the Soil Species Records of Yunnan Province, the samples in this study included nine soil types: Lateritic Red Soil, Red Soil, Dry Red Soil, Purple Soil, Paddy Soil, Laterite, Cinder Soil, Yellow Soil, and Yellow Brown Soil. We term this variable “soil type”.

Based on land use patterns, the habitats of the sampling points were categorized into three types: farmland, vegetable garden, and forest. We defined this as the habitat type variable.

### 2.2. Nucleic Acid Extraction, ddPCR Assay, and Positive Determination

Each soil sample was accurately weighed at 1.5 g, and nucleic acids were extracted using a commercial kit: DNeasy PowerSoil Pro Kit (QIAGEN GmbH, Hilden, Germany Cat. No.: 175041867), yielding 70 μL of nucleic acid extract. The concentration and purity of the extracted genomic DNA were measured. DNA concentration was quantified using a Qubit fluorometer (Thermo Fisher Scientific, Waltham, MA, USA), with a concentration of ≥10 ng/µL considered acceptable. DNA purity was assessed using a Biodrop micro-spectrophotometer, and samples with A260/A280 ratios between 1.7 and 2.0, and A260/A230 ratios ≥ 1.8 were deemed qualified.

The Bio-Rad QX200 ddPCR (Bio-Rad Laboratories Inc., Hercules, CA, USA) system (equipped with two fluorescence channels) was used in the Droplet Digital PCR (ddPCR) assay. A duplex ddPCR detection kit for *Yersinia pestis* (Cat. No.: dPCR-II-406), provided by Zhuhai Huirui Biotechnology Co., Ltd. (Zhuhai, China), was employed. The primer and probe sequences for the target genes were detailed in Table S1. In this assay, the *caf1* gene was labeled with FAM fluorescence, and the *ypo0392* gene was labeled with VIC fluorescence. The sequences of the probes and primers contained in the kit are presented in Table S1. The total reaction volume was 20 µL. The reaction components, their final concentrations, and added volumes were as follows: 5 µL of 4× dPCR Probe Master Mix (Bio-Rad Laboratories Inc., Hercules, CA, USA) was used at a final concentration of 1×; primers (*caf1*-FP, *caf1*-RP, *ypo0392*-FP, and *ypo0392*-RP), each with an initial stock concentration of 100 µM, were added at 0.1 µL each; probes (*caf1*-P and *ypo0392*-P), each with an initial stock concentration of 100 µM, were added at 0.05 µL each; 10 µL of the DNA template was added; and the reaction mixture was brought to a final 20 µL volume with nuclease-free water.

The thermal cycling conditions for the ddPCR reaction were as follows: an initial enzyme activation step at 96 °C for 10 min, followed by 40 amplification cycles, each consisting of denaturation at 94 °C for 30 s and annealing/extension at 55 °C for 1 min, and a final enzyme deactivation step at 98 °C for 10 min. After the reaction, the QX200 system was set to hold at 4 °C for 30 min. The thermal lid temperature was maintained at 105 °C throughout the entire process.

The positive control was a 150-fold diluted genomic DNA extract of *Yersinia pestis*, prepared from 1.2 mL of bacterial suspension at McFarland concentration 6.5 using the High Pure PCR Template Preparation Kit (Roche (Basel, Switzerland), Cat. No. 11796828001RCH), with a final volume of 100 µL. Sterile nuclease-free water was used as a blank control.

The raw data were obtained as copies per µL and copies per reaction. Copy number per reaction was used for positivity determination. The limit of detection (LoD) for *caf1* was set at 8.9 copies/reaction, and the LoD for *ypo0392* at 15.4 copies/reaction. The LoD values used (8.9 copies/reaction for *caf1* and 15.4 for *ypo0392*) were derived from a previous study using the same instrument and similar targets [13]. When both *ypo0392* and *caf1* or *ypo0392* were detected, the result was determined as positive.

### 2.3. Statistical Analysis

The distribution of *Yersinia pestis* nucleic acids was described using the positivity rate, while the abundance of *Yersinia pestis* nucleic acids was expressed as the copy number per gram of soil (derived from copy number per reaction).

The raw data on the positivity rate were binary categorical data, and for the positivity rates, Fisher’s exact test or the chi-square test for trend was applied. If significant, Fisher’s exact test with Bonferroni correction was used for post hoc pairwise comparisons.

Copy numbers were continuous variables; box plots were used to describe their central tendency and dispersion. To compare copy numbers of two independent samples, the Mann–Whitney U test was used, and to compare multiple samples, the Kruskal–Wallis H test was used. If significant, Dunn’s test with Bonferroni correction was used for post hoc pairwise comparisons. All statistical analyses were performed in the R version 4.5.0 programming environment, with the significance level set at α = 0.05.

## 3. Results

From August 2024 to May 2025, a total of 78 soil samples were collected from 16 counties in Yunnan Province. Among them, 51 samples were from plague foci and 27 from non-foci. The samples spanned nine soil types, with collection numbers ranging from three to 13 per type, and three habitat types (farmland, vegetable garden, and forest), with 37, 16, and 25 samples collected, respectively. See Table S2 for details.

### 3.1. Results of ddPCR

**Figure 1:**
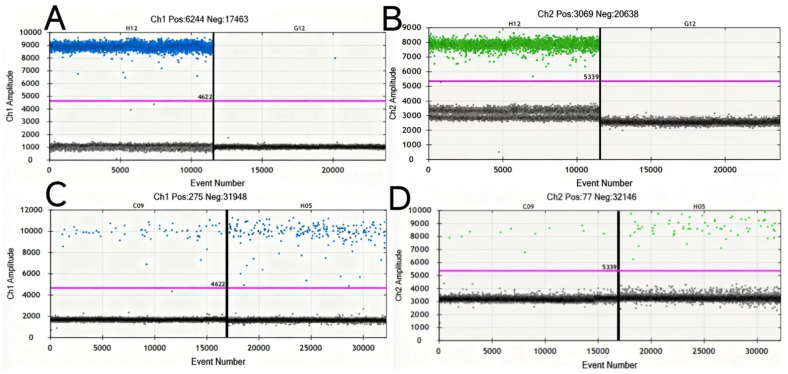
The original one-dimensional scatter plot of the ddPCR results. In the figure, the blue droplets represent FAM fluorescence-labeled droplets, indicating detection of *caf1*; the green droplets represent VIC fluorescence-labeled droplets, indicating detection of *ypo0392*; the black droplets show no fluorescent signal, representing negative droplets; and the purple lines indicate the threshold separating positive and negative droplets. Well H12 is a positive control, and well G12 is a negative control. Well C09 is a sample that reaches the limit of detection (LoD) in the FAM channel, with the corresponding sample ID YM-8. Well H05 is a sample that reaches the LoD in both channels, with the corresponding sample ID JH-33. Panels (**A**,**B**) show the FAM and VIC fluorescence detection results for the positive and negative controls. Panels (**C**,**D**) show the FAM and VIC fluorescence detection results for the samples.

The positive control yielded expected copy numbers in both FAM and VIC channels, while the blank control showed no amplification. The mean effective total droplet number across all samples was 17,667. The droplet clusters were well-separated, and the boundaries between positive and negative droplets were clearly defined. See Figure 1 for details.

### 3.2. Nucleic Acid Positivity Rates of Soil Samples and Statistical Analyses

Among these 78 soil samples, nucleic acid ddPCR detection showed that the copy number of the *caf1* gene exceeded the limit of detection (LoD, 8.9 copies/reaction) in 22 samples, and that of the *ypo0392* gene exceeded the LoD (15.4 copies/reaction) in 13 samples. A total of 13 samples had both genes above the LoD and were determined as positive for *Yersinia pestis*. We specifically list the sampling information and gene copy number of the samples determined as positive in Table 1.

The overall nucleic acid positivity rate was 16.67% (13/78). By epidemiological type, the positivity rate was 15.7% (8/51) in plague foci and 18.5% (5/27) in non-plague foci. By soil type, the positivity rates were as follows: Red Soil 9.1% (1/11), Lateritic Red Soil 23.1% (3/13), Dry Red Soil 50.0% (2/4), Yellow Soil 0% (0/10), Yellow Brown Soil 0% (0/10), Purple Soil 60.0% (3/5), Laterite 100.0% (3/3), Paddy Soil 7.70% (1/13), and Cinder Soil 0% (0/9). By habitat type, the positivity rates were 27.0% (10/37) for farmland, 12.5% (2/16) for vegetable garden, and 4.0% (1/25) for forest. See Table S2 for details.

The positivity rates of soil nucleic acid among the three grouping variables (epidemiological type, soil type, and habitat type) were statistically compared using Fisher’s exact test. The results showed that only the soil type group exhibited a statistically significant difference (*p* < 0.001). Subsequently, pairwise comparisons were performed for the soil types using Fisher’s exact test with Bonferroni correction for multiple testing. After correction, all pairwise *p*-values were greater than 0.05, indicating no statistically significant differences between any two soil types. See Table 2 for details.

### 3.3. Copy Numbers of Soil Samples and Statistical Analyses

In terms of copy number, the median for the FAM channel (*caf1*) was 5.60 (the unit is copies per gram of soil, the same below, and is omitted hereafter), with an interquartile range of 196.00, and a range spanning from 0.00 to 1558.66. For the VIC channel (*ypo0392*), the median was 0.00, with an interquartile range of 41.30, and a range from 0.0000 to 476.00. These results indicate that both the level and variability in copy numbers were higher in the *caf1* compared to *ypo0392*.

**Figure 2:**
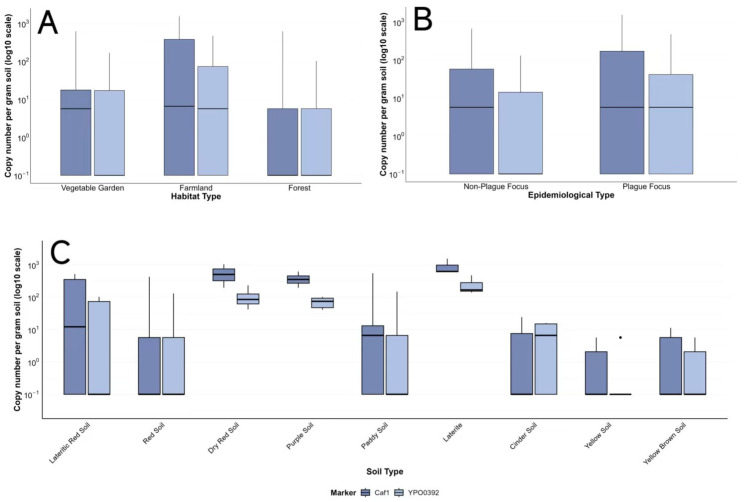
Boxplots of gene copy numbers. The vertical axis refers to copy number [log_10_(copy number/gram soil)] of *caf1* and *ypo0392*, and the horizontal axis refers to the specific categories of the grouping variables. (**A**): habitat type, (**B**): epidemiological type, and (**C**): soil type. The single data point for Yellow Soil (*ypo0392*) represents an outlier. It indicates that this data point differs from the others and may warrant attention, but it is not necessarily an error.

Two fluorescence channels were involved in the copy number analysis: the FAM channel corresponded to gene *caf1*, and the VIC channel corresponded to gene *ypo0392*. The copy numbers of the two genes were expressed as copies per gram of soil. Since most copy numbers spanned more than three orders of magnitude, a Log10 transformation was applied. The distribution of the transformed data is presented as a boxplot, as shown in Figure 2. The median copy number of *caf1* was generally close to or higher than that of *ypo0392*. In the epidemiological type, the median copy number for *ypo0392* was lower in non-plague foci than in plague foci. In the soil type, the median copy numbers of the three soil types (Purple Soil, Dry Red Soil, and Laterite) were significantly higher than those of the other categories.

For statistical comparison of copy numbers of *caf1* and *ypo0392* across three grouping variables, the Mann–Whitney U test was used for the epidemiological type variable group, and the Kruskal–Wallis H test was used for the soil type and habitat type variable groups. The overall comparison showed that *caf1* and *ypo0392* in the soil type group and *ypo0392* in the habitat type group had *p*-values < 0.05. Subsequently, pairwise comparisons were performed for these variable groups using Dunn’s test and Bonferroni adjustment. The results showed that in the soil type variable group for *caf1*, among the 36 pairwise comparisons, five pairs (e.g., Yellow Soil–Purple Soil, Yellow Soil–Dry Red Soil, Yellow Soil–Laterite, Yellow Brown Soil–Dry Red Soil, and Yellow Brown Soil–Laterite) had *p*-values < 0.05. For *ypo0392*, among the 36 pairwise comparisons, six pairs had *p*-values < 0.05, including an additional pair (Yellow Brown Soil–Purple Soil) compared to *caf1*. In the habitat type variable group, for *ypo0392*, one out of three pairwise comparisons (i.e., forest–farmland) had a *p*-value < 0.05. Details are shown in Table 3.

## 4. Discussion

This study aimed to detect the presence of *Yersinia pestis* signals in soil from natural plague foci, so as to provide insights into the scientific question of “where *Yersinia pestis* goes during the quiescent phase of the plague”. We used droplet digital PCR (ddPCR) combined with a soil-specific nucleic acid extraction kit to detect *Yersinia pestis* nucleic acid amplification signals. The detection sensitivity of ddPCR is higher than that of real-time quantitative PCR (qPCR) [13], and the dedicated extraction kit effectively removes substances that inhibit PCR reactions in soil [15]. Our sampling design considered multi-dimensional sources of the samples, including plague foci and non-foci areas, nine soil types, and three land use patterns. The objectives were, first, to determine whether *Yersinia pestis* signals exist in the soil, and second, to assess the correlations between *Yersinia pestis* presence and the epidemiological type, soil type, and habitat type. Contrary to our expectations, positive *Yersinia pestis* signals were also detected in non-focus areas. Moreover, no correlation was observed between distribution and abundance of *Yersinia pestis* signals relative to the classification of an area as a plague focus or non-focus region.

### 4.1. The Significance of Studying Soil

Plague alternates between epidemic and quiescent phases; however, as a natural focus disease, plague will not disappear as long as the natural focus exists. The mechanism by which *Yersinia pestis* is maintained during quiescent periods remains unresolved. A plague natural focus is an ecosystem that sustains the long-term persistence of *Y. pestis* in nature, with core components including the pathogen, hosts, vectors, and the geographical environment suitable for their survival. Based on this, the present study took soil—a tangible component of the geographical environment—as the research object. In the sampling design, only the soil depth was fixed, while burrow soil of rodents was deliberately excluded. The underlying reason was to allow the collected soil to exist independently of host and vector influences, thereby better reflecting the geographical environment as an independent factor. *Y. pestis* nucleic acids had a long persistence capacity under certain conditions in the environment and could be maintained for months or even thousands of years, particularly under low-temperature conditions or within closed structures, such as protozoan cysts [16] and ancient dental pulp [17,18,19].

In soil and burrow soil samples from plague-endemic areas in Kazakhstan, the presence of *Y. pestis* was detected through biochemical identification [20]. After inoculating virulent *Y. pestis* into sterilized soil, the pathogen remained detectable for 40 weeks. A molecular historical review of plague explicitly stated that *Y. pestis* could persist in soil for several months, serving as a source of re-infection for burrowing mammals. The existence of a soil reservoir complemented the traditional rodent–flea transmission model and provided a new mechanism to explain the reactivation of plague natural foci after prolonged silence [21]. These studies collectively suggested that soil may serve as a natural reservoir of plague.

However, dissenting views hold that *Y. pestis* can survive for at least 24 days in naturally contaminated soil, as shown in an investigation of human plague cases [22]. A large-scale environmental survey of soil and aerosol samples across multiple locations in the United States tested for four pathogens, including *Y. pestis* and its close relatives. The results showed that *Y. pestis* was detected at low frequencies in environmental samples, and no sample tested positive for both phylogenetic and virulence genes simultaneously. Nevertheless, close relatives or related sequences of *Y. pestis* were found to be distributed in soil [23].

### 4.2. Advantages of ddPCR for Detecting Nucleic Acids in Soil

ddPCR is capable of detecting nucleic acid molecules down to single-copy level, offering significant advantages in low-abundance pathogen detection, trace residual pathogen monitoring, and rare mutation detection [24,25,26]. Multiple studies have confirmed that ddPCR outperforms qPCR in inhibitor-rich samples, such as soil, feces, and wastewater, exhibiting higher sensitivity and lower false-negative rates [12,13,16]. Because ddPCR partitions the reaction mixture into a large number of independent droplets, the inhibitory effect of these substances on PCR amplification is significantly reduced [27]. The detection limit of ddPCR in soil could reach 10^2^ CFU per 100 mg of soil, and its sensitivity in simulated soil samples was 10 times higher than that of qPCR [13,14]. By partitioning the reaction mixture into tens of thousands of droplets, and directly calculating the absolute copy number of target nucleic acids based on a Poisson distribution, ddPCR eliminated the need for a standard curve [24,28], thereby avoiding the inter-batch variation and quantification uncertainty associated with standard curve construction in qPCR. This made ddPCR particularly suitable for environmental samples that lack reference materials [12].

The *caf1* gene is a virulence gene located on the plasmid of *Y. pestis* that encodes the F1 antigen; this plasmid is one of the key acquired genetic elements distinguishing *Y. pestis* from *Y. pseudotuberculosis* [13]. The *ypo0392* gene is a chromosomal marker gene specific to *Y. pestis*. The dual-target detection technique targeting *caf1* and *ypo0392* has played an important role in confirming human plague cases in Inner Mongolia [29] and Yunnan [5], as well as in confirming wild rodent plague outbreaks in Yunnan, China [30]. This indicates that the application of dual-*caf1* and *ypo0392* as detection targets has a reliable basis. Environmental plasmids were generally present at low abundance in soil bacterial communities, making their detection challenging [31]. However, in the present study, the *caf1* gene was more readily detected than the *ypo0392* gene. Because the *caf1* gene was located on a plasmid—an extrachromosomal genetic element—we assigned greater weight to the *ypo0392* gene when determining positivity. During bacterial replication, plasmids generally exist in higher copy numbers than chromosomal genes. So, if both *ypo0392* and *caf1* or *ypo0392* were detected, the result was determined as positive. Coincidentally, in all samples where the copy number of *ypo0392* exceeded its limit of detection (LoD), the copy number of *caf1* also exceeded its LoD, further indicating that *ypo0392* carried greater weight between the two targets.

The positive determination of ddPCR directly depends on the limit of detection (LoD): a sample can only be determined as positive when the concentration of the target is equal to or higher than the LoD. If the concentration is lower than the LoD, it is determined as negative or below the detection limit. The LoD of ddPCR is closely related to the total number of droplets generated. Ideally, more than 10,000 valid droplets are required to ensure reliable detection of low-abundance targets [12,24]. In this study, the Bio-Rad QX200 ddPCR system produced 17,000 valid droplets under a reaction volume of 20 μL.

### 4.3. The Relationship Between Soil Y. pestis Positivity and Epidemic Focus

This study examines the relationship between the detection of *Y. pestis* signals in soil and the presence of epidemic foci. In this study, epidemic foci refer to locations where *Y. pestis* has been previously isolated, whereas non-epidemic foci refer to locations where *Y. pestis* has never been isolated. Therefore, according to conventional thinking, the detection of *Y. pestis* amplification signals in soils from non-epidemic foci is unexpected. It is worth noting that the long-standing detection of plague outbreaks has primarily targeted hosts and vectors, from which *Y. pestis* has also been isolated. In contrast, soil, as an important component of the geographical environment, has long been neglected. The detection of *p* signals in soils from both epidemic and non-epidemic foci, with no statistically significant difference between them, prompts new considerations regarding our understanding of the environmental persistence mechanisms of *Y. pestis*.

### 4.4. The Relationship Between Soil Y. pestis Positivity and Soil Type

In this study, the statistical analysis for the grouping variable “soil type” was more complex than those for the other variables. There is no statistical difference in positivity rate, but there is a statistical difference in copy number. The positivity rate reflected qualitative detection, whereas the copy number represented quantitative detection, indicating a qualitative–quantitative decoupling. The primary reason for this discrepancy is that the positivity rate is dichotomized based on a threshold, leading to over-aggregation of intergroup differences and substantial loss of internal information. In contrast, the copy number, as a continuous variable, fully retains and utilizes all data information, thereby capturing substantive intergroup differences that remain masked below the threshold. Given that the copy number provided more comprehensive information, we concluded that soil type did have a certain influence on the detection of *Y. pestis* nucleic acid amplification signals.

Our study found that among the nine soil types examined, three (Purple Soil, Dry Red Soil, and Laterite) exhibited higher copy numbers. According to the classification principles outlined in “Soil Species in Yunnan”, the soil classification systematically integrated soil-forming conditions, pedogenetic processes, and soil properties among various soil types [32]. This implies that each soil type is a product shaped by long-term interactions between specific bio-climatic conditions. Its current properties (e.g., pH value, mineral composition, and organic matter content) represent the ultimate outcome of its unique pedogenetic processes. Purple soils have distinct physicochemical properties compared with other soil types. By analyzing environmental data (soil characteristics and climate) from active plague foci in China and extrapolating to Europe, it was found that soil texture and biochemistry were two factors that make Europe less conducive to the existence of long-term plague foci [33]. Although the sample sizes for these three soil types were small in our study, the box plots show that their copy numbers not only had high medians but also small interquartile ranges, indicating a high degree of data concentration. This suggests that these soil types should be the focus of future research.

### 4.5. Relationship Between Soil Y. pestis Positivity and Soil Habitat Type

Similar to soil types, habitat types also exhibited qualitative–quantitative decoupling. Land-use types shaped soil bacterial communities through changes in soil physicochemical properties [34]. Vegetation type and land use patterns can exert complex effects on microbial communities by altering soil properties. Tillage practices had a particularly critical impact on soil microorganisms [35]. Frequent anthropogenic disturbances in cultivated land (e.g., irrigation, fertilization, or straw incorporation) can increase the relative abundance of soil bacterial and fungal communities. Meanwhile, stable communities in forestland may exhibit stronger biological resistance to invading pathogens. These studies all point to the general principle that “habitat type shapes the distribution pattern of microorganisms.”

### 4.6. Study Limitations and Prospects

First, the limit of detection (LoD) for *caf1* and *ypo0392* served as the threshold for determining *Y. pestis* positivity. Determining the LoD typically requires experiments such as parallel testing of blank samples and low-concentration gradient dilution assays; however, this study did not perform such experiments but instead referred to values from the literature. Fortunately, the digital PCR instrument used in the referenced literature was of the same brand and model as that used in this study. Moreover, one of the detection targets was the same *caf1*. Although the second target was not *ypo0392*, it was also a specific gene located on the chromosome. Therefore, the LoD from the literature met the conditions to serve as a reference. Future work should include a formal LoD determination using matrix-matched controls.

Second, this study was a subproject of a larger research project, and the sample size design was constrained by the overall layout of the main project. As a result, the sample sizes for certain subgroups (e.g., specific soil types) were relatively small, which may affect the statistical power of the tests.

Third, the soil samples that tested positive for *Y. pestis* would have offered an opportunity to isolate viable bacteria; however, this study did not carry out such isolation.

Soil is an integral part of the geographical environment, and other environmental matrices, such as water bodies, air media, and vegetation, also warrant further investigation. These may provide broader perspectives for understanding the mechanisms underlying the prevalence and maintenance of *Y. pestis* and even other natural focal diseases.

## 5. Conclusions

The copy number of *Y. pestis* nucleic acids detected was a comprehensively informative indicator, capable of capturing subtle changes in information more sensitively than the positivity rate. A certain correlation existed between soil type and *Y. pestis* signals. To delve into the persistence mechanisms of plague, it is necessary to move beyond the constraints of the traditional natural focus definition. As a crucial component of natural foci, soil deserves greater attention. This study represents only a preliminary exploration of soil, and more in-depth and systematic investigations will be carried out in the future.

## Figures and Tables

**Table 1:** Sampling information and gene copy number of soil samples positive for Yersinia pestis tested by ddPCR.

No.	Sample ID	Sampling Location	Epidemiological Type	Soil Type	Habitat Type	*caf1* Copy Number/μL	*ypo0392* Copy Number/μL	*caf1* Copy Number/Reaction	*ypo0392* Copy Number/Reaction	*caf1* Copy Number/Gram Soil	*ypo0392* Copy Number/ Gram Soil	Determination of *Y. pestis*
1	FQ-25	Fengqing	Plague Focus	Lateritic Red Soil	Farmland	5.5	0.79	110	15.8	513.33	73.73	‘+
2	NJ-38	Nanjian	Non-Plague Focus	Red Soil	Farmland	4.6	1.4	92	28	429.33	130.66	‘+
3	YM-10	Yuanmou	Non-Plague Focus	Dry Red Soil	Farmland	7.2	1.1	144	22	672	102.66	‘+
4	LF-11	Lufeng	Non-Plague Focus	Purple Soil	Farmland	4.9	1	98	20	457.33	93.33	‘+
5	LF-19	Lufeng	Non-Plague Focus	Purple Soil	Forest	6.7	1.1	134	22	625.33	102.66	‘+
6	LF-20	Lufeng	Non-Plague Focus	Purple Soil	Farmland	3.8	0.8	76	16	354.66	74.66	‘+
7	ZY-10	Zhenyuan	Plague Focus	Paddy Soil	Farmland	5.9	1.6	118	32	550.66	149.33	‘+
8	MJ-16	Mojiang	Plague Focus	Lateritic Red Soil	Vegetable Garden	4.1	1	82	20	382.66	93.33	‘+
9	MJ-17	Mojiang	Plague Focus	Lateritic Red Soil	Farmland	5.6	1.1	112	22	522.66	102.66	‘+
10	YJ-22	Yuanjiang	Plague Focus	Dry Red Soil	Farmland	11.2	2.5	224	50	1045.33	233.33	‘+
11	JH-32	Jinghong	Plague Focus	Laterite	Farmland	6.5	1.5	130	30	606.66	140	‘+
12	JH-33	Jinghong	Plague Focus	Laterite	Farmland	16.7	5.1	334	102	1558.66	476	‘+
13	JH-34	Jinghong	Plague Focus	Laterite	Vegetable Garden	6.7	1.8	134	36	625.33	168	‘+

**Table 2:** Statistical comparison of positive rates among different grouping variables.

Grouping Variable	Analysis Level 1	Statistical Method 1	*p*-Value	Analysis Level 2	Statistical Method 2	*p*-Value (Corrected)
Epidemiological Type(2)	Overall	Fisher’s exact	0.758	NA		
Soil Type(9)	Overall	Fisher’s exact	<0.001	Pairwise	Fisher’s exact & Bonferroni adjustment	Totaling 36 comparisons, all corrected *p*-values were greater than 0.05.
Habitat Type(3)	Overall	Fisher’s exact	0.056	NA		

Note: Statistical significance was determined at the threshold of *p* < 0.05. The details of the pairwise comparisons were shown in Table S3.

**Table 3:** Statistical comparison of copy number among different grouping variables.

Grouping Variable	Gene	Analysis Level 1	Statistical Method 1	*p*-Value	Analysis Level 2	Statistical Method 2	*p*-Value (Corrected)
Epidemiological Type(2)	*caf1*	Overall	Mann–Whitney U test	0.603	NA		
	*ypo0392*	Overall	Mann–Whitney U test	0.372	NA		
Soil Type(9)	*caf1*	Overall	Kruskal–Wallis H test	<0.001	Pairwise	Dunn’s test & Bonferroni adjustment	Of the 36 pairwise comparisons, those with *p* < 0.05 are as follows: Yellow Soil–Purple Soil, Yellow Soil–Dry Red Soil, Yellow Soil–Laterite, Yellow Brown Soil–Dry Red Soil, Yellow Brown Soil–Laterite.
	*ypo0392*	Overall	Kruskal–Wallis H test	<0.001	Pairwise	Dunn‘s test & Bonferroni adjustment	Of the 36 pairwise comparisons, those with *p* < 0.05 are as follows: Yellow Soil–Purple Soil, Yellow Soil–Dry Red Soil, Yellow Soil–Laterite, Yellow Brown Soil–Dry Red Soil, Yellow Brown Soil–Laterite, Yellow Brown Soil–Purple Soil.
Habitat Type(3)	*caf1*	Overall	Kruskal–Wallis H test	0.09	NA		
	*ypo0392*	Overall	Kruskal–Wallis H test	0.036	Pairwise	Dunn‘s test & Bonferroni adjustment	Of the 3 pairwise comparisons, those with *p* < 0.05 are as follows: Forest–Farmland.

Note: Statistical significance was determined at the threshold of *p* < 0.05. The details of the pairwise comparisons are shown in Tables S4–S6.

## Data Availability

The original data supporting the conclusions of this study are included in the Supplementary Materials of the article. All relevant datasets, including soil sample information, nucleic acid copy number detection results, and statistical analysis data, are detailed in Supplementary Materials, which are accessible as part of the published manuscript. Further inquiries regarding the data can be directed to the corresponding authors.

## Supplementary Materials

The following supporting information can be downloaded at: https://www.mdpi.com/article/10.3390/pathogens15060616/s1, Table S1. Primers and probes for detecting *Y. pestis*. Table S2. Information of soil samples and ddPCR copy numbers. Table S3. Pairwise comparisons of positive rates among different soil types. Table S4. Statistical comparison of *carf1* copy number among different soil types. Table S5. Statistical comparison of *ypo0392* copy number among different soil types. Table S6. Statistical comparison of *ypo0392* copy number among different habitat types.
